# Case of Renal Calculus

**Published:** 1857-03

**Authors:** Charles M. Hewitt

**Affiliations:** Geneva, N. Y.


ART. V.— *Case of Renal Calculus.* By CHARLES M. HEWITT, M. D., Geneva, N. Y.

I send you the history of a case of renal calculus, which, if you consider it of sufficient interest, you are at liberty to insert in your Journal. The case occurred in the practice of Dr. J. Smith, of this village, who has kindly furnished me with the particulars of the patient's history embodied in this report:

Miss S., a maiden lady, æt. 57. She traced the beginning of her difficulties to a strain of the back which she received twelve years ago.

She had been under treatment at various times since for an excessive irritability of the stomach, which so increased that about two years previous to her death, she was unable to eat any but the lightest food, and that in the smallest quantity. This state of things continued until her death.

Six weeks before that time she called Dr. Smith's attention to a swelling in the right lumbar region, which, she said, she had not thought of enough importance to tell him of, though she had noticed it for years. He found it to be about the size of a child's head, and there being evident fluctuation, punctured it, and drew off a pint and a-half of pus. The relief was but temporary. The patient died shortly after. I extract the account of the post-mortem from my note book:

"Patient much emaciated. Opened abdomen by verical incision. The tumor, which extended from the liver to the middle of right iliac, was adherent to the anterior walls of the abdomen, the muscular portions of which were at that point absorbed. The cavity of the abscess from which the Dr. drew the pus, was found external to the tumor, and of the size of a dollar. The ascending colon and portions of the small intestines, were adherent to the inner side of the diseased mass, and the colon just above the adhesion, was diminished to the size of one's little finger, for several inches.

"Having dissected off these attachments and elevated the tumor, it was found to be the enlarged pelvis of the right kidney. The body of the kidney was entire, with the exception of the lower portion, which was absorbed into the diseased mass. The contents of the tumor were pus mixed with a cheesy kind of matter, and over the entrance to the ureter was a calculus, irregular as to surface and outline, but about an inch square. In the infundibula and extending into the pelvis of the kidney, we found another, three inches and one half long, one inch wide, having on one side a projecting portion an inch long and wide. The thickness was from one-quarter to onehalf an inch. Its texture is firm, and its color almost white. It seems to have been deposited in layers, which are evident in the specimen.

"I should think that the tumor would contain nearly two pints of pus. The left kidney was healthy and of usual size. The fundus of the uterus was occupied by a tumor of scirrhus appearance, and smaller ones of the same kind were found in other parts of the organ, and in the ovaries. One extremity of the pancreas was a little indurated.

"The calculi are principally composed of phosphate of lime. The weight of the large one is just one ounce."

